# A Case of Invasive Sinonasal Carcinosarcoma: The Importance of Early Detection

**DOI:** 10.1155/2018/2745973

**Published:** 2018-04-22

**Authors:** Jason Yuen, Vinay Varadarajan, Marios Stavrakas, Samiul Muquit, Hisham Khalil

**Affiliations:** ^1^South West Neurosurgery Centre, Derriford Hospital, Plymouth PL6 8DH, UK; ^2^Department of Ear, Nose and Throat, Derriford Hospital, Plymouth PL6 8DH, UK

## Abstract

Sinonasal carcinosarcomas represent rare neoplasms, with aggressive character and unfavourable prognosis. We present a case of extensive sinonasal carcinosarcoma extending into the anterior cranial fossa and into the orbit and also a review of the current international literature regarding this rare yet aggressive neoplasm. There is currently a lack of specific guidelines on the optimal management of sinonasal carcinosarcoma and the treatment represents a challenge for the clinician. The key message that we would like to disseminate to our colleagues is the importance of suspicion and early detection, as well as the necessity to adopt a holistic approach when counselling patients.

## 1. Introduction

A malignant biphasic neoplasm consisting of an epithelial (squamous) element and mesenchymal component is known as a carcinosarcoma (also known as pleomorphic carcinoma, spindle cell carcinoma, pseudosarcoma, and pseudosarcomatous squamous cell carcinoma) [1, 2] although the exact nomenclature and subclassifications are variable [3]. It is classified under squamous cell carcinoma by the World Health Organization (WHO) [4].

Carcinosarcoma may arise from any squamous epithelium (e.g., salivary glands, respiratory tract, upper aerodigestive tract, and female reproductive organs) [5], but its occurrence in the sinonasal region is extremely rare [1]. There is very little evidence available about its best management.

## 2. Case Presentation

A 75-year-old gentleman first presented to primary care with a three month history of left-sided headaches and diplopia. He also complained of altered taste sensation and paraesthesia in the left maxillary region. There was no history of epistaxis. The patient attended his general practitioner on multiple occasions and was given sinusitis treatment until a CT scan was finally performed due to persistence of symptoms, upon which the patient was then referred to the ENT service.

He has a background history of fast atrial flutter (on bisoprolol), hypertension, polymyalgia rheumatica, benign prostatic hyperplasia, and previously excised papillary squamous cell carcinoma in the left thigh. He quit smoking 40 years prior to presentation. Otherwise, he had lived independently with WHO performance status of 1 (Karnofsky status 90).

The patient underwent complete head and neck examination including flexible nasoendoscopy. There was no discreet neck lymphadenopathy. Nasoendoscopy revealed a mass down to the left inferior turbinate obscuring the left nasal cavity. He complained of diplopia on the left side on the lateral gaze with proptosis of approximately 3 mm compared to the contralateral side. Vision was 6/30 bilaterally with glasses. There was no relative afferent pupillary defect or papilloedema.

### 2.1. Investigations

Initial CT scan revealed an aggressive lesion in left ethmoidal and frontal sinuses, invading the left orbit and anterior cranial fossa. CT neck and thorax showed no cervical or chest lymphadenopathy.

**Figure 1:**
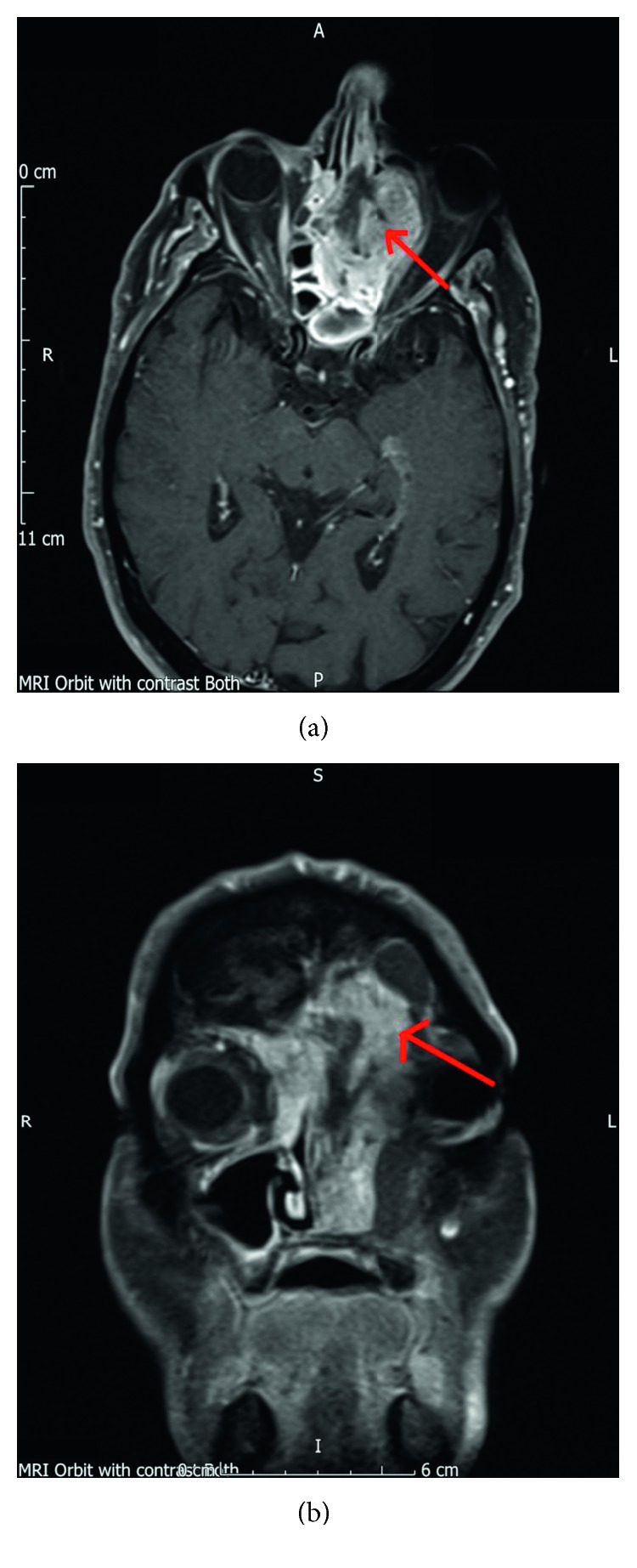
MRI head scan shows breaching of dura mater. Red arrow denotes lesion of interest. (a) Axial slide. (b) Coronal slide.

Subsequent MRI imaging also showed evidence of bone erosion with breaching of the dura in the vicinity of the left orbitofrontal cortex although there was no signal change in the brain to suggest brain invasion (Figure 1). There was destruction of left lamina papyracea. An incidental right anterior cranial fossa meningioma, distant from sinonasal lesion was also identified. This pathologic finding did not have any significant clinical relevance with the primary disease.

A staged whole body positron-emission tomography (PET) scan showed no other distant lesions but confirmed lesion progression through the frontal sinus.

Our initial differential diagnoses were squamous cell carcinoma, carcinosarcoma, lymphoma, teratocarcinosarcoma, olfactory neuroblastoma, small cell carcinoma and alveolar rhabdomyosarcoma.

### 2.2. Treatment

After the diagnostic workup, the patient underwent endoscopic examination of the nose and biopsy of the lesion under general anaesthesia. Extensive disease was noted at the ipsilateral maxillary antrum. Histology revealed an extensive necrotic biphasic epithelioid, spindled malignant neoplasm in keeping with carcinosarcoma. Immunohistochemistry afterwards showed no loss of DNA mismatch repair (MMR) protein expression.

### 2.3. Outcome

After a successful biopsy and radiological investigative workup, the patient was discharged home. Unfortunately in the community, his preexisting comorbidities worsened, and he developed poorly controlled fast atrial flutter and urosepsis with E. Coli bacteraemia. He was readmitted for antimicrobial and supportive treatment. The patient eventually recovered and was discharged.

Discussion was undertaken at the multidisciplinary meeting and with international experts in the field. The consensus is that although the disease can potentially be resected with major surgery such as craniofacial resection, due to its location and size, surgical treatment would have significant risk of bleeding, cerebrospinal fluid (CSF) leak, and meningitis. After detailed consultation and discussion about the possible treatment options including the risks of such procedures with the patient and his family, he was not keen to proceed and opted for palliative radiotherapy and symptomatic management.

The radiotherapy dose delivered was 20 Gy in five days, then a two-week break and finally another 20 Gy in five days. One year after diagnosis, patient tolerated the radiotherapy and continued to lead an independent life.

## 3. Discussion

In the head and neck area, carcinosarcomas most frequently occur in the larynx and oral cavity, followed by the skin, tonsils, sinonasal tract, pharynx and hypopharynx [6, 7]. Histologically, they have been classified as part of a spectrum of sarcomatoid carcinomas, most of which present in late middle-aged men with a long history of tobacco use. They consist of foci of overt carcinoma admixed with areas of divergent differentiation into mesenchymal tissues [8]. Spindle cell formation is also typical in the sarcomatous component. A recent study suggested that a MET protooncogene mutation may be a prerequisite event in its pathogenesis [9].

Presenting symptoms of sinonasal carcinosarcomas typically include nasal obstruction, epistaxis, facial pain, and headache [10, 11]. These are rather nonspecific to the disease. They tend to grow rapidly, with extensive local destruction. Therefore, early diagnosis and aggressive therapy are necessary to improve the often dismal prognosis [2, 8].

In a Japanese case report of maxillary carcinosarcoma, the patient died despite intensive radiochemotherapy and total maxillectomy due to rapid tumour recurrence and metastasis. However, it appeared the carinomatous component responded to the radiochemotherapy but that was not the case with the sarcomatous component [11].

Nonetheless, aggressive treatment may not cease disease progression and improve survival. Cheong et al. [12] reported on a 61-year-old male patient who underwent total maxillectomy and modified radical neck dissection and died shortly afterwards with sternal metastasis, despite having had extensive surgery with curative intent.

Table 1 shows the similar cases of sinonasal carcinosarcoma reported in the literature [1, 2, 9–31]. The optimal treatment of this disease remains undetermined. It is difficult to ascertain the effectiveness of the treatment modalities (a combination of surgery, radiotherapy, and chemotherapy) from the small number of cases available in the literature. They generally confer a poor outcome and a high recurrence rate. A recent large case-control analysis of 15 sinonasal patients in America showed an average five-year disease-specific survival of 48.5%, which is significantly poorer than controls with carcinosarcoma at other anatomical sites [5]. Therefore we advocate, when patients are operatively fit with resectable disease, they should undergo aggressive surgical treatment with adjuvant radiotherapy as an attempt to improve outcome. This is because radiotherapy alone tends to convey a less favourable outcome, and the role of chemotherapy is unclear.

Since the optimal management of carcinosarcoma remains uncertain, it is intuitive to study a related but separate (also highly aggressive) entity known as teratocarcinosarcoma [26, 33–35]. This tumour type also includes a component of neuroectodermal tissue and is much more prevalent in the medical literature. According to a recent systemic review of 49 patients, it is recommended that optimal treatment involves radical surgical resection followed by radiation therapy [25].

If more doctors are aware of this condition and the associated presenting symptoms, perhaps earlier diagnoses can be made. Therefore, patients may potentially get the option of a curative total resection, since the invasion of the skull base and surrounding structures is a poor prognostic factor.

In conclusion, sinonasal carcinosarcomas represent rare neoplasms, with aggressive character and unfavourable prognosis. Here we present a case of extensive sinonasal carcinosarcoma extending into the anterior cranial fossa and into the orbit and also a review of the current international literature regarding this rare yet aggressive neoplasm. There is currently a lack of specific guidelines on the optimal management of sinonasal carcinosarcoma, and the treatment represents a challenge for the clinicians. The key message that we would like to disseminate to our colleagues is the importance of suspicion and early detection, as well as the necessity to adopt a holistic approach when counselling patients.

## Figures and Tables

**Table 1:** Cases of sinonasal carcinosarcoma that are reported in the literature.

Number	Age (years)/sex	Location	Stage	Treatment	Outcome	Author, year
1	62/F	Maxillary sinus	ND	RT	No marked improvement of the tumor, DWD	Meyer and Shklar, 1957 [24]
2	71/M	Maxillary + ethmoid sinuses	T4aN0M0	Preoperative RT + TM + RE	ND about postoperative tumor condition, death due to intracerebral abscess at postoperative period	Feinmesser et al., 1982 [16]
3	65/F	Maxillary sinus	ND	TM + RT	LR, DWD 8 months later	Ampil, 1985 [14]
4	57/F, (postradiation)	Nasoethmoid sinus	ND	Tumor excision, ethmoidectomy and turbinectomy	LR 5 months after surgery, outcome uncertain	Hafiz et al., 1987 [17]
5	60/M	Nasomaxillary sinus	T3N0M0	TM + RT + CT	LR, DWD 2 months later (diffuse metastasis in lung and brain)	Sonobe, 1989 [11]
6	53/M	Maxillary + ethmoid sinuses	T4aN0M0	TM + craniofacial resection + RT + CT	Disease free after 9 months	Shindo et al., 1990 [2]
7	81/F	Maxillary sinus	T3N0M0	TM + RT + 2nd operation	LR, DWD 3 months after second operation	Sanabre et al., 1998 [27]
8	47/M	Maxillary sinus	ND	PM+RT	LR, DWD after 1 year	Furuta et al., 2001 [10]
9	54/M	Maxillary sinus	T3N3M0	RT + CT	DWD 4 months; possibly from lung metastasis	Howard et al., 2007 [19]
10 case series (19 cases)	Mean age at diagnosis 54 (range, 42–66) Male 11 Female 8	Sinonasal track, unclear exact locations	T1/2 3 T3/4 16 N0 19 M0 19	Surgery alone 2 Surgery and RT 7, RT alone 2 CT + RT 2	Mean follow-up 38 months (6–40) 5 with disease at last follow-up 0 death at follow-up	Doshi et al., 2010 [15]
11	75/M	Nasal cavity	T4N0M0	Surgery + CT + RT	Disease-free after 5 years	Terada and Kawasaki, 2011 [30]
12	60/M	Maxillary sinus and nasal cavity	Unclear	CT + RT	DWD after 9 months	Terada, 2011 [29]
13	29/F	Nasopharyngeal	T1N2cM0	RT	Clinically free-of-disease 2 months after RT	Lim et al., 2012 [22]
14	60/M	Maxillary + ethmoid sinuses	T3N0M0	TM + RT + CT	LR, FL	Moon, 2013 [1]
15	29/M	Nasal cavity	T2N0M0	Surgery	Disease-free after 6 months	Gupta, 2013 [32]
16	52/M	Frontal, sphenoid, ethmoid, and maxillary sinuses	T4aN0M0	TM + RT + CT	LR	Alem and AlNoury, 2014 [13]
17	61/M	Maxillary sinus	T4aN2cM1	TM with modified neck dissection	DWD shortly after surgery with sternal metastasis	Cheong et al., 2014 [12]
18 case series (15 cases)	Mean age at diagnosis 60.3 (SD, ±21.3) Male 6 Female 9	Nasal cavity 7 Maxillary sinus 5 Ethmoid sinus 1 Frontal sinus 1 Sphenoid sinus 1	T1/2 3 T3/4 10 TX 2 N0 9 N+ 0 NX 6 M0 14 M1 0 MX 1	Surgery alone 3 surgery and RT 7 RT alone 4 No therapy 1	Five-year disease-specific survival of 48.5%	Patel et al., 2015 [5]
19	66/M	Maxillary sinus	T3N0M0	PM + RT + CT	LR, DWD 10 months after initial presentation	Ando et al., 2015 [9]
20	78/F	Ethmoid sinus	T4N0M0	Surgery + RT	Disease-free after 36 months	Iqbal et al., 2015 [20]
21	66/M	Maxillary sinus	Recurrence	Palliative	DWD after 12 months	
22	68/M	Maxillary sinus	Recurrence	Palliative	DWD after 1 month	
23	46/M	Maxillary sinus	T4N0M0	Palliative	DWD after 5 months	
24	54/M	Sphenoid sinus	T4 N and M unclear	Surgery + CT + RT	DWD after 12 months	Liu et al., 2016 [23]
25	55/M	Maxillary sinus	T4aN0M0	RT	No significant effect, died 4 months after initial examination	Hasnaoui et al., 2017 [18]
26	35/F	Maxillary sinus	T4N0M0	PM + CT + RT	DWD after 12 months	Soltani et al., 2018 [28]
27	75/M	Ethmoid + frontal sinuses	T4aN0M0	RT	Remained independent after 12 months	Our case, 2018

ND, not described; RT, radiation therapy; DWD, dead with disease; TM, total maxillectomy; RE, removal of eye; LR, local recurrence; CT, chemotherapy; PM, partial maxillectomy; FL, follow-up loss; LNs, lymph nodes.
